# Impairments in goal-directed action and reversal learning in a proportion of individuals with psychosis

**DOI:** 10.3758/s13415-022-01026-8

**Published:** 2022-08-02

**Authors:** Shuichi Suetani, Andrea Baker, Kelly Garner, Peter Cosgrove, Matilda Mackay-Sim, Dan Siskind, Graham K Murray, James G Scott, James P. Kesby

**Affiliations:** 1 Queensland Centre for Mental Health Research, Brisbane, QLD 4076 Australia; 2 Queensland Brain Institute, The University of Queensland, Brisbane, QLD 4072 Australia; 3 School of Medicine and Dentistry, Griffith University, Brisbane, QLD 4111 Australia; 4 Institute for Urban Indigenous Health, Brisbane, QLD 4030 Australia; 5 Centre for Human Brain Health, School of Psychology, University of Birmingham, B0121 Birmingham, UK; 6 Metro North Mental Health, Royal Brisbane and Women’s Hospital, Brisbane, QLD 4029 Australia; 7 Metro South Addiction and Mental Health Services, Brisbane, QLD 4102 Australia; 8 Faculty of Medicine, University of Queensland, QLD 4072 Brisbane, Australia; 9 Department of Psychiatry, University of Cambridge, Cambridge, UK; 10 Cambridgeshire and Peterborough NHS Foundation Trust, Cambridge, UK; 11 Institute for Molecular Bioscience, University of Queensland, Brisbane, Australia; 12 QIMR Berghofer Medical Research Institute, Brisbane, QLD 4029 Australia

**Keywords:** Schizophrenia, Schizoaffective, Behavior, Decision-making, Basal ganglia

## Abstract

Cognitive impairment in psychosis is one of the strongest predictors of functional decline. Problems with decision-making processes, such as goal-directed action and reversal learning, can reflect cortico-striatal dysfunction. The heterogenous symptoms and neurobiology observed in those with psychosis suggests that specific cognitive phenotypes may reflect differing causative mechanisms. As such, decision-making performance could identify subgroups of individuals with more severe cortico-striatal dysfunction and help to predict their functional decline. The present work evaluated the relationship between goal-directed action, reversal learning, and symptom profiles in those with psychosis. We assessed decision-making processes in healthy controls (N = 34) and those with persistent psychosis (N = 45), subclassifying subjects based on intact/impaired goal-directed action. Compared with healthy controls (<20%), a large proportion (58%) of those with persistent psychosis displayed impaired goal-directed action, predicting poor serial reversal learning performance. Computational approaches indicated that those with impaired goal-directed action had a decreased capacity to rapidly update their prior beliefs in the face of changing contingencies. Impaired decision-making also was associated with reduced levels of grandiosity and increased problems with abstract thinking. These findings suggest that prominent decision-making deficits, indicative of cortico-striatal dysfunction, are present in a large proportion of people with persistent psychosis. Moreover, these impairments would have significant functional implications in terms of planning and abstract thinking.

**Supplementary Information:**

The online version contains supplementary material available at 10.3758/s13415-022-01026-8.

## Introduction

Cognitive impairments are strong predictors of functional outcomes across psychiatric diagnoses (Crouse et al., 2020), particularly for those living with psychosis (Hochberger et al., 2020). People with psychosis suffer from a range of cognitive impairments, including deficits in working memory, verbal/visual learning, reasoning, and problem solving (Marder, 2006). Therefore, there is a strong interest in identifying cognitive features that help to predict illness trajectories for these individuals (Nelson et al., 2017; Reichenberg et al., 2010).

Psychotic disorders are heterogeneous in both neurobiology and symptom profile. However, robust evidence highlights a strong link between psychosis and subcortical dopamine systems (Kesby et al., 2018; Li et al., 2020). For example, positron emission tomography (PET) studies have demonstrated that excessive dopamine signaling in the associative striatum underlies psychotic symptoms and potentially cognitive deficits (Conn et al., 2020; Ersche et al., 2011; McCutcheon et al., 2018). The associative striatum receives a rich set of connections from higher-order cortical regions and selectively gates incoming cortical information (Conn et al., 2020). This enables associative striatal networks to modulate information flow to generate and adapt responses for action selection (i.e., decision-making) (Sharpe et al., 2018). Moreover, dysfunctional cortico-striatal circuits may underlie the specific decision-making problems that are common in those with psychotic disorders (Adida et al., 2011; Bates et al., 2002; Chudasama & Robbins, 2006; Kesby et al., 2021; Morris et al., 2018; Pantelis et al., 2004). After dopamine stimulation and in psychosis (Clatworthy et al., 2009; Dandash et al., 2014; Morris et al., 2015; Sarpal et al., 2015), changes in the functional connectivity and activation of the associative striatum are evident, suggesting that these circuits have a causative role in decision-making deficits.

Decision-making involves the contribution of a range of brain areas and circuits. We have proposed that two tests—outcome-specific devaluation and serial reversal learning—represent a functional readout that is sensitive to associative striatal dysfunction (Conn et al., 2020; Kesby et al., 2018). Outcome-specific devaluation requires the participant to learn two action-outcome associations, after which one outcome is devalued. When given the choice between the two actions after devaluation, healthy controls respond more toward the valued outcome over the devalued. This shows the ability to adapt to newly acquired information (Morris et al., 2015; Morris et al., 2018). Altered activation of the associative striatum (caudate) has been reported to underlie impairments in outcome devaluation in people with schizophrenia (Morris et al., 2015). Reversal learning tasks require the participant to adapt when two outcomes are continuously reversed. This is generally conducted in a probabilistic environment, where one stimulus has a high reward probability (80%) and the other has a low reward probability (20%) (Izquierdo et al., 2017). We also use computational modeling and fit reinforcement learning algorithms to each subject’s sequence of choices (Ahn et al., 2017; den Ouden et al., 2013), providing insight into the cognitive processes that drive behavior. In reversal-learning studies, increases in the associative striatal dopamine levels of healthy individuals have been shown to correlate with decreased performance (Clatworthy et al., 2009), with deficits in reversal learning also linked to thought disorder in schizophrenia (Pantelis et al., 2004). With overlapping corticostriatal regulation, whether deficits in outcome-specific devaluation predict a specific phenotype in reversal learning has not been investigated in those with psychosis. Moreover, it represents a novel translational approach to investigate associative striatal dysfunction in preclinical models (Kesby et al., 2018).

The goals of the present study were 1) to establish whether people with persistent psychosis display deficits in both outcome devaluation and reversal learning compared with healthy individuals, and 2) to assess whether deficits in outcome devaluation are associated with specific reversal learning phenotypes.

## Participants and methods

### Participants

A total of 79 participants, between 18-50 years of age, were classified into two groups: those diagnosed with a psychotic disorder (N = 45) and healthy controls (no diagnosis of a psychotic disorder and had not experienced a psychotic episode; N = 34). The general psychiatric characteristics for those with psychosis are presented in Table S1. Detailed inclusion criteria are described in the Supplementary Methods.

### Procedures and experimental design

All procedures were approved by the Royal Brisbane and Women’s Hospital and University of Queensland Human Research Ethics Committees (HREC/17/QRBW/168). Participants gave informed consent, and their anonymity was preserved. Participants were remunerated $40AUD (see Supplementary Methods). Premorbid and current IQ was assessed by using the Test of Premorbid Functioning (TOPF; Pearson Clinical, Sydney, Australia) and Wechsler Abbreviated Scale of Intelligence, second edition (WASI-II; Pearson Clinical). Substance use was assessed using a Substance Misuse Scale (Duhig et al., 2015). Psychosis symptoms were assessed with the Positive and Negative Syndrome Scale [PANSS]. The cognitive tasks were run using PsychoPy v3 (Peirce et al., 2019) with stimuli being displayed on a computer monitor. Responses were recorded on a joystick box (Fighting stick mini 4; Hori Co. Ltd, Yokohama, Japan).

### Outcome-specific devaluation task

**Fig. 1:**
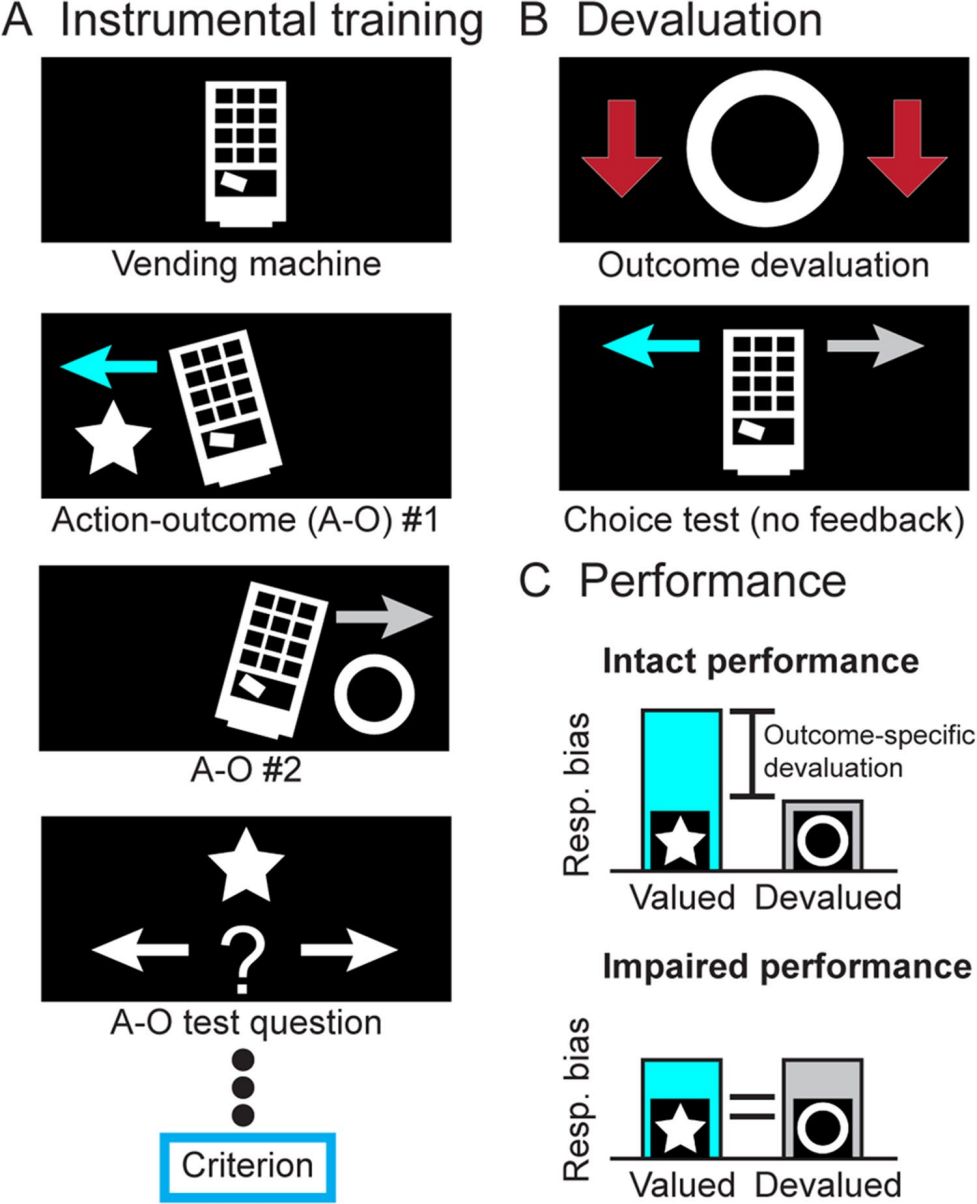
**Outcome-specific devaluation task.** In this task, participants are trained to learn two action-outcome (A-O) associations by tilting a vending machine left or right with a joystick (**A**). To confirm that they have learned these relationships, a test question is presented after three stimulus presentations. Participants must answer six consecutive test questions correct to reach criterion. Following instrumental training, participants are explicitly informed that one outcome is now worth fewer credits (outcome devaluation) and undergo a choice test (**B**). The choice test is a fixed period during which the participant can tilt the vending machine left or right at will but receives no feedback (requiring them to remember the causal association between the action and outcome, in lieu of the outcome). Performance is assessed using the responses (resp.) and response bias between the valued and devalued actions (**C**). A significant bias in responding toward the valued outcome (intact performance) indicates intact goal-directed action, whereas a lack of this bias indicates impaired performance

We used an outcome devaluation task (Fig. 1) adapted from Morris et al. (Morris et al., 2015).

#### Instrumental training

Participants were told that three tokens (visual stimuli) were of equal value. Using a 7-point Likert scale, participants rated each token (Fig. S1) based on how valuable they considered it to be and their motivation to earn tokens. Training involved liberating two of the tokens from a virtual vending machine. The joystick was moved left or right, with 5-10 consecutive responses (drawn randomly) in one direction required to earn the associated token (e.g., star or circle). After every three rounds, a question was posed to assess participants’ understanding of the association between action and reward. After getting six questions correct in a row, instrumental training ended.

#### Devaluation test

Participants were informed that one of the tokens had been counterfeited (counterbalanced) and was therefore less valuable (making this token the devalued option). Participants were instructed to tilt the vending machine to earn the associated tokens and were told that their actions in this stage would dictate their monetary compensation. The virtual machine was displayed for 10 blocks (12 seconds) and could be tilted at will during each block. Aside from visually tilting the vending machine, no feedback was presented. This forces participants to use action-outcome associations rather than stimulus-outcome responses. Subsequently, participants were asked probe questions about which outcome was associated with which action and rerated the value of each token and their motivation to receive tokens.

### Serial-reversal learning task

**Fig. 2:**
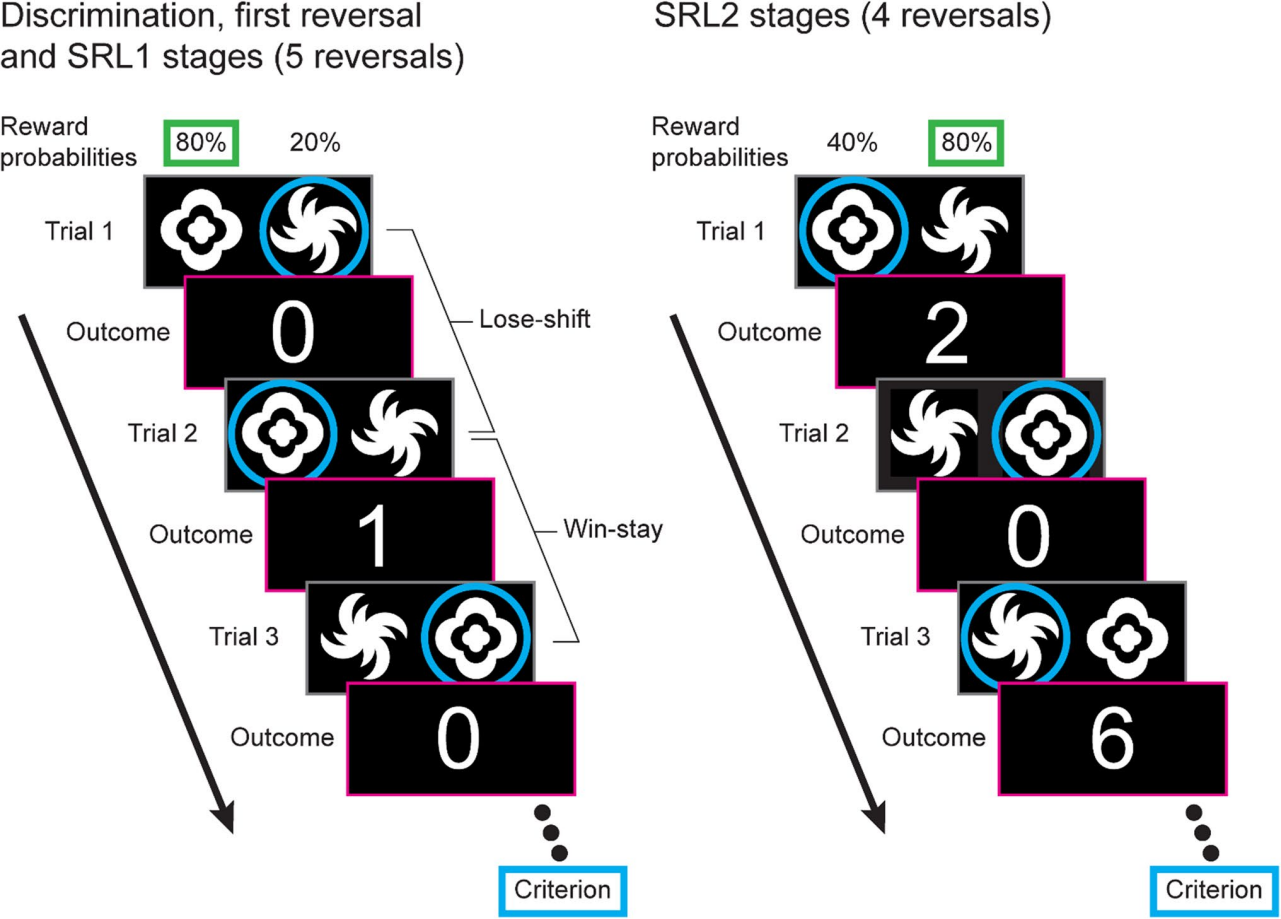
**Serial-reversal learning task.** For each trial participants are presented with two stimuli (which remain the same throughout the entire task). For the initial discrimination, first reversal, and serial-reversal learning (SRL) 1 stages (left), these are probabilistically rewarded with one stimulus rewarded on 80% of trials (cloud-like stimulus in this example) and the other at 20% (spiral-like stimulus in this example). After selecting a stimulus (blue circle), the credit reward is displayed on the screen (0 or 1 credit). A Lose-shift is recorded when the participant changes the alternative stimulus after a loss (i.e., after a loss on Trial 1, 0 credits, the other stimulus is selected for Trial 2), and a Win-stay is recorded when the participant selects the same stimulus after a win (i.e., Trial 2 wins 1 credit, and the same stimulus is selected for Trial 3). Criterion is reached when six consecutive choices of the higher probability stimulus are made. For the next stage, the stimulus probabilities are reversed. For the SRL2 stages (right), the probability of receiving a reward on the poorer stimulus is increased to 40% and participants can receive either 2 or 6 credits for a win (equal probability)

For the reversal learning task (Fig. 2), all stimulus pairs were binary images (Fig. S2), and all combinations were counterbalanced (for detailed methodology and training stages see Supplementary Methods).

#### Probabilistic reversal learning

Participants underwent a probabilistic reversal learning task consisting of 11 stages; initial discrimination (1 stage), initial reversal (1 stage), and serial reversal learning phase 1 (SRL1; 5 stages) and phase 2 (SRL2; 4 stages). Each featured the same pair of stimuli but with variations in the reward rate (probabilistic) and outcome (credits). For the first seven stages, the probabilistic reward contingencies were set at 80/20, meaning that the target stimulus was rewarded 80% of the time, and the nontarget stimulus was rewarded 20% of the time. One credit was earned for a rewarded trial and 0 credits for a nonrewarded trial. For the SRL2 stages, the contingencies were set at 80/40, increasing the task difficulty by providing more misleading feedback. Two or six credits were earned for a rewarded trial (equal probability) and 0 credits for a nonrewarded trial. Criterion for progressing from each stage was 6 correct responses in a row.

#### Reversal learning performance measures and strategies

General performance measures included total trials to criterion, perseveration (number of errors in the first 6 trials after a reversal), and response rates. Whether a subject selected the same stimulus after attaining a reward (Win-stay) or selected the alternative stimulus after a loss (Lose-shift) was quantified as a proportion of the total applicable trials.

### Computational modeling and simulation

The underlying cognitive processes in reversal learning were calculated by modeling latent task variables using the hBayesDM package for R (version 3.6 [Platform: x86_64-w64-mingw32/x64 (64-bit)] on Windows 10 v1809) developed by Ahn et al. (Ahn et al., 2017). An experience-weighted attraction model (EWA) that had previously shown a good fit to reversal learning behavior and association with dopaminergic polymorphisms was examined (den Ouden et al., 2013). Parameters included learning rate (*phi*), experience decay (*rho*; how quickly prior information is updated), and inverse temperature (*beta*; the deterministic or exploratory nature of the choices made).

To establish which parameters were responsible for alterations in performance, we simulated performance after the manipulation of each individual parameter to generate “hypothetical” outcomes based on the model-driven performance (N = 20/group).

### Data analysis

Binary variables were examined by using χ^2^ tests, and continuous variables used analysis of variance (ANOVA) with *Group* as the independent variable (with repeated measures where necessary). The discrimination ratio and response rates for outcome devaluation also were analyzed using within-group paired *t* tests to confirm significant goal-directed action. To classify subjects as having intact or impaired goal-directed action we performed hierarchical clustering analyses using Ward’s method and Squared Euclidean distance. Variables (preference ratio and response rates for the valued action on outcome devaluation) were transformed using Z scores. Computational simulations were first compared using ANOVAs and then to control simulations using the Dunnett's test for multiple comparisons. All statistical analyses were performed with IBM SPSS Statistics 26 (Armonk, NY). When appropriate, post-hoc comparisons were performed using Šídák corrections. Results are expressed as mean ± standard error of the mean (SEM). Differences were considered statistically significant at *p* < 0.05. Preference and response bias figures were made with code adapted from (van Langen, 2020).

## Results

### Decision-making in group with persistent psychosis

Compared with controls, persistent psychosis subjects had significantly fewer years of education and a lower average premorbid and current IQ (See Table S2 for demographics, IQ and substance use). The persistent psychosis group also had a higher level of lifetime use for multiple substances.

### Goal-directed action is impaired in a large proportion of those with persistent psychosis

**Fig. 3:**
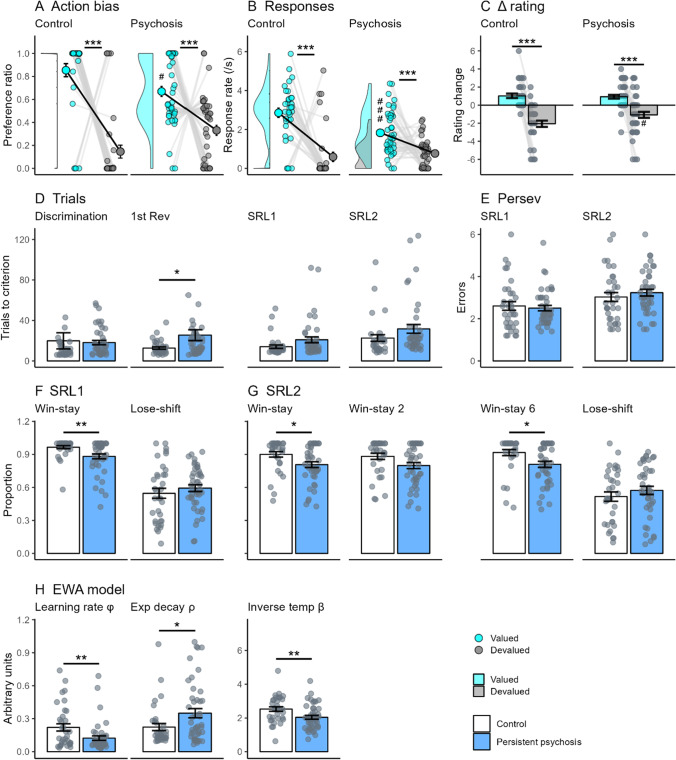
**Decision-making performance in those with persistent psychosis.** Comparison of outcome devaluation and reversal learning performance in healthy controls and those with persistent psychosis. For outcome devaluation, persistent psychosis subjects showed a significant bias (**A**) and response (**B**) toward the valued outcome (aqua) after devaluation (**C**). However, the preference toward the valued outcome was significantly less than that observed in controls and featured a bimodal frequency distribution as seen in the frequency histogram along the Y axis (**A**). For serial-reversal learning, persistent psychosis subjects (blue) took significantly more trials to reach criterion (**D**) for the first reversal and trended toward the same for the SRL1 and SRL2 stages. These increases were not associated with changes in the number of perseverative errors (**E**). The strategies used for the SRL1 (**F**) and SRL2 (**G**) showed a similar pattern, with persistent psychosis subjects using fewer Win-stays than control subjects. Differences in computational modeling parameters were observed for all parameters in the EWA model (**H**). Data are displayed as the mean ± standard error. *p < 0.05; **p < 0.01; ***p < 0.001; ^#^*p* < 0.05; ^###^*p*<0.001 compared with controls

Our analyses revealed that both the control and persistent psychosis groups significantly biased their preference (Fig. 3A) and their rate of responding (Fig. 3B) toward the valued response. However, both the preference (*F*_2,78_ = 6.9, *p* < 0.05) and rate of responding (*F*_2,78_ = 12.8, *p* < 0.001) for the valued response was significantly lower in persistent psychosis subjects compared with controls (for all comparisons see Table S3). These data confirm previous reports that persistent psychosis subjects have deficits in goal-directed action (Morris et al., 2015; Morris et al., 2018); however, the bimodal response bias (Fig. 3A) suggests that this impairment is not observed in all individuals.

When we examined the decreases in goal-directed action, we found that these were not due to changes in reward valuation. Both groups showed a significant decrease in their rating for the devalued versus the valued outcomes after devaluation (Fig. 3C; controls, *t*_33_ = 8.0, *p* < 0.001; persistent psychosis, *t*_43_ = 5.6, *p* < 0.001). There was no significant difference between groups in the average number of correct responses for the probe questions, indicating that they both recollected the action-outcome associations equally.

### Persistent psychosis subjects switch more after rewards in serial-reversal learning

Goal-directed action requires the understanding that outcome values have changed and acting accordingly, whereas serial-reversal learning requires the constant updating of uncertain outcome values based on trial-by-trial experience. There were significant differences between groups for the trials to criterion in the first reversal (*F*_1,76_ = 4.3, *p* < 0.05), and a trend in the SRL1 stage (*F*_1,76_ = 3.8, *p* = 0.056), due to an increase in the average trials required for the persistent psychosis group compared with controls (Fig. 3D; for all comparisons see Table S4). These were not accompanied by alterations in the number of perseverative errors (Fig. 3E). The proportion of Win-stay, but not Lose-shift, use was significantly different between groups in the SRL1 stage (Fig. 3F; *F*_1,76_ = 9.0, *p* < 0.01) and the SRL2 stage (Fig. 3G; *F*_1,69_ = 5.4, *p* < 0.05). Those with persistent psychosis used Win-stay strategies less than controls, particularly after winning a 6 in the SRL2 stage (*F*_1,69_ = 6.2, *p* < 0.05).

Computational modeling indicated that a range of decision-making processes were different between persistent psychosis subjects and controls. For the EWA model (Fig. 3H), there were significant differences between groups for all parameters, with persistent psychosis subjects having a lower learning rate (*F*_1,76_ = 7.2, *p* < 0.01), higher experience decay (*F*_1,76_ = 4.6, *p* < 0.05), and lower inverse temperature (*F*_1,76_ = 8.6, *p* < 0.01). Lower learning rate values in the EWA model (1-learning rate) indicate that increased prediction error signaling biased learning toward using more recent information. This would appear to be incongruent with the observed decrease in Win-stay use. However, the EWA learning rate does not distinguish between reward and loss learning. Therefore, we also used a reward/punishment model (Fig. S3; learning parameters were inverted to match the EWA parameter). This model confirmed the observed decrease in inverse temperature in those with persistent psychosis (*F*_1,76_ = 12.8, *p* < 0.001), in addition to lower punishment learning (*F*_1,76_ = 5.0, *p* < 0.05) and higher reward learning (*F*_1,76_ = 4.7, *p* < 0.05). The substantial decrease in punishment learning rate (>40%) indicates that changes in the EWA learning rate parameter in those with psychosis are likely driven by a change in the response to loss, with a bias toward learning from more recent losses than controls. Altered punishment learning is unlikely to impact Win-stay use, but higher reward learning would decrease Win-stay use (i.e., lower impact of the most recent win). However, the difference between controls and those with persistent psychosis was modest, suggesting other cognitive processes underlie this outcome (as we demonstrate with our simulations in Fig. 5). For example, lower inverse temperature values reflect less deterministic or more exploratory decision-making, which would decrease Win-stay use (i.e., more chance of shifting after a win to explore the alternative stimulus). Higher experience decay values indicate a slower decay or updating of experience weight with changing contingencies. Thus, multiple cognitive processes are altered in those with persistent psychosis, but on balance this appears to selectively alter the response to recent rewards (i.e., Win-stay) in the context of reversal learning.

### Large proportion of those with persistent psychosis have broad decision-making deficits

Given the bimodal distribution for the valued lever preference in the persistent psychosis group (Fig. 3A), we used hierarchical clustering analyses to classify each group into intact and impaired goal-directed action subgroups (Fig. 1C). The cluster analysis separated the cohort based on a response bias of ~0.77, with 0.81 the lowest bias in the intact psychosis group and 0.72 the maximum in the impaired psychosis group. This point corresponded well with the minimum point between the peak distributions in those with persistent psychosis (Fig. 3A). Based on this split, the proportion of impaired subjects was greatest in the persistent psychosis group (controls, 28 intact/6 impaired; persistent psychosis, 18 intact/25 impaired). As low numbers prevented control comparisons (see Table S5 for control intact/impaired data), analyses included the control intact (n = 25), and persistent psychosis intact (n = 18) and impaired (n = 25) subgroups. The demographical and psychiatric information for these groups can be found in Tables S6 and S7.

### Impaired goal-directed action is not due to impaired reward valuation

**Fig. 4:**
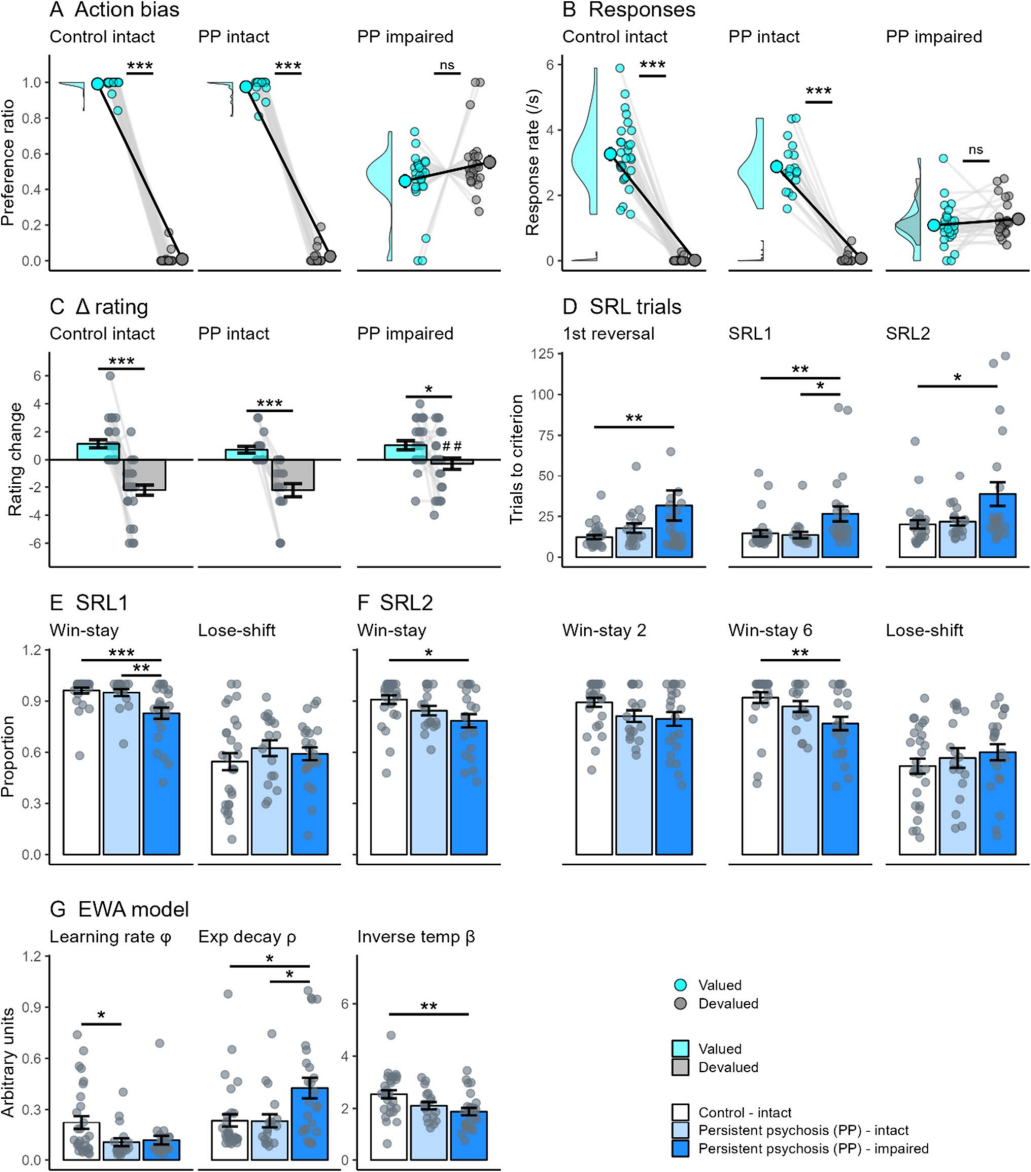
**Intact and impaired goal-directed action subgroups in those with persistent psychosis**. Comparison of performance in the outcome devaluation and reversal learning tasks in subjects classified as control and persistent psychosis (PP) with intact goal-directed action, and persistent psychosis with impaired goal-directed action. For outcome devaluation, persistent psychosis subjects with impaired goal-directed action did not show a preference towards the valued outcome (aqua) after devaluation (**A** and **B**). Persistent psychosis subjects with impaired goal-directed action (dark blue) showed a significant change in their rating of the valued and devalued outcomes, highlighting that they were aware of the change in reward value (**C**). For serial-reversal learning, persistent psychosis subjects with impaired goal-directed action took more trials to reach criterion (**D**) than control (white) and persistent psychosis groups with intact goal-directed action (light blue). This was likely a consequence of a significant reduction in Win-Stay use during both the SRL1 (**E**) and SRL2 stages (**F**). Differences in computational modeling parameters were observed in the EWA model (**G**). Decreased learning rate (*phi*) and inverse temperature (*beta*) values were observed in persistent psychosis groups. A significant increase in experience decay (*rho*) values in persistent psychosis subjects with impaired goal-directed action compared with both the other groups was the only cognitive process that differentiated the persistent psychosis groups. Data are displayed as the mean ± standard error. *p < 0.05; **p < 0.01; ***p < 0.001; ^##^*p* < 0.01 compared with the equivalent measure in controls

Figure 4A and B show the preference and response rates for subgroups with intact and impaired goal-directed action (for all comparisons see Table S8). All groups showed a significant reduction in their rating for the devalued compared with valued outcomes after devaluation, indicating that impairments in reward valuation do not underlie impaired goal-directed action (Fig. 4C). There was a significant difference between groups in the magnitude of change for the devalued outcome (*F*_2,68_ = 7.8, *p* < 0.001). Persistent psychosis subjects with impaired goal-directed action had a smaller decrease in rating compared with both other groups (*p* < 0.01). However, differences in the level of devaluation were independent of deficits in goal-directed action (see Table S9 for performance when matched for level of devaluation). There were no significant differences between groups in the average number of correct responses for the probe questions following devaluation, indicating that all groups recollected the action-outcome associations.

### A decreased capacity to respond to contingency changes underlies reversal learning deficits in persistent psychosis subjects with impaired goal-directed action

We were interested in whether deficits in goal-directed action were associated with specific reversal learning phenotypes, because these may indicate shared neurobiology. Overall, general performance deficits in reversal learning were limited to persistent psychosis subjects with impaired goal-directed action, suggesting that this subgroup has broad decision-making problems. These also were specific to reversal learning (for all comparisons see Table S10), with no significant differences between groups in the trials to criterion for the initial discrimination. There were significant differences in the average trials to criterion for the first reversal (*F*_2,68_ = 3.3, *p* < 0.05), the SRL1 stage (*F*_2,68_ = 6.3, *p* < 0.01), and the SRL2 stage (*F*_2,61_ = 4.5, *p* < 0.05) (Fig. 4D). The persistent psychosis subjects with impaired goal-directed action took more trials than the control group for the first reversal (*p* < 0.05) and the SRL2 stage (*p* < 0.05). For the SRL1 stage, the persistent psychosis subjects with impaired goal-directed action took more trials than both other groups (*p* < 0.05). Significant differences in the proportion of Win-stay strategy use were evident during the SRL1 (Fig. 4E; *F*_2,68_ = 9.6, *p* < 0.001) and SRL2 stages (Fig. 4F; *F*_2,64_ = 4.1, *p* < 0.05). Persistent psychosis subjects with impaired goal-directed action had a significantly lower Win-stay use on the SRL1 stage than both other groups (*p* < 0.01). For the SRL2 stage, changes in Win-stay use between controls and the persistent psychosis subjects with impaired goal-directed action were greatest after winning a 6 (*p* < 0.01).

Computational modeling highlighted that performance deficits in persistent psychosis subjects with impaired goal-directed action were associated with a unique combination of impaired processes. There were significant differences between groups for all parameters of the EWA model (Fig. 4G). Differences in the learning rate parameter (*F*_2,68_ = 4.2, *p* < 0.05) were driven by significant decreases in the persistent psychosis subjects with *intact* goal-directed action compared with the controls (*p* < 0.05) and a trend toward the same in the persistent psychosis subjects with impaired goal-directed action (*p* = 0.052). Differences in the experience decay parameter (*F*_2,68_ = 5.6, *p* < 0.01) were driven by a significant increase in the persistent psychosis subjects with impaired goal-directed action compared with both other groups (*p* < 0.05). Differences in the inverse temperature parameter (*F*_2,68_ = 5.8, *p* < 0.01) were driven by a significant decrease in the persistent psychosis subjects with impaired goal-directed action compared with controls (*p* < 0.01). Therefore, sluggish updating of experience weighting with changing contingencies (experience decay) appear to underlie the reversal-specific performance deficits in persistent psychosis subjects with impaired goal-directed action.

### Sluggish experience updating and less deterministic choices underlie deficits in persistent psychosis subjects with impaired goal-directed action

**Fig. 5:**
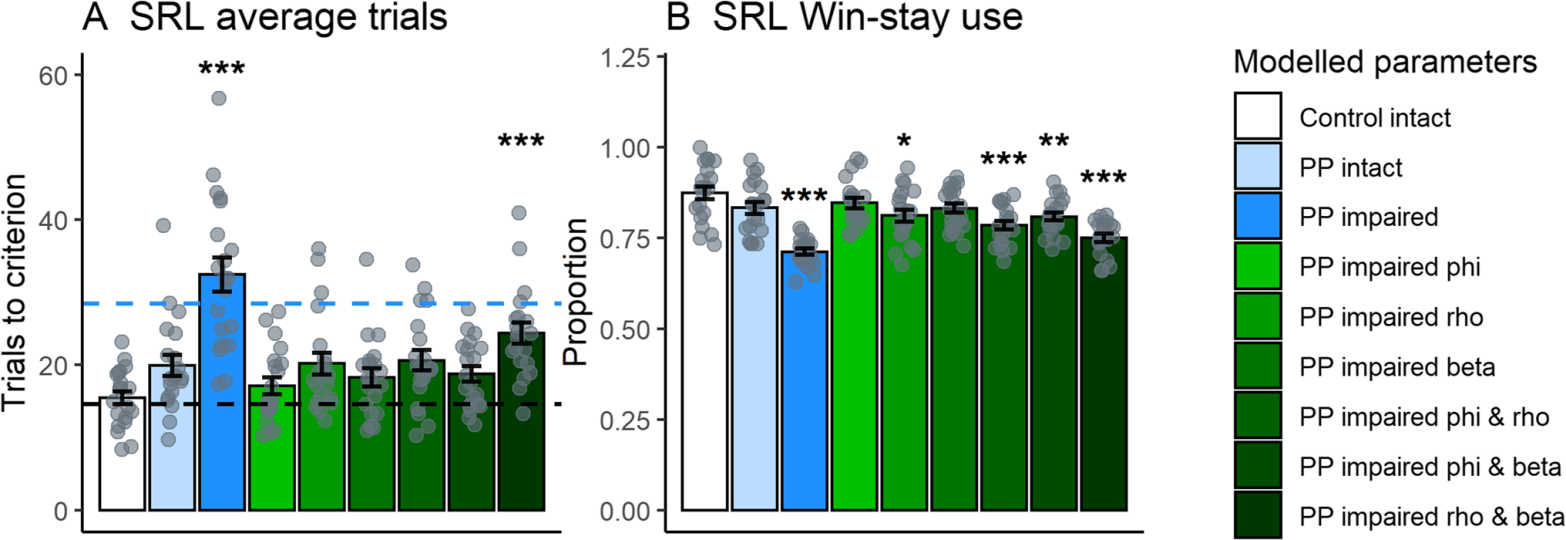
**Computational simulations of decision-making performance in subgroups with intact and impaired goal-directed action.** Simulations were run using the extracted parameters from the EWA model for controls with intact goal-directed action (Control intact [white]), and persistent psychosis subjects with intact (PP intact [light blue]) and impaired (PP impaired [dark blue]) goal-directed action. Compared with the control simulations, persistent psychosis subjects with impaired goal-directed action simulations took more average trials to reach criterion in the SRL stages (**A**) and used Win-stay strategies less (**B**). The simulated data provided a good reflection of the real performance, demonstrated by the similar number of trials to reach criterion in the SRL1 stages (black and blue dotted lines reflect actual control intact and PP impaired averages, respectively). The green bars represent control intact parameters with one or two values substituted for PP impaired values (i.e., for light green, PP impaired *phi* with Control intact *rho* and *beta*). Only the combination of PP impaired *rho* and *beta* values (darkest green) led to both an increase in SRL trials to criterion and reduced Win-stay use. Data are displayed as the mean ± standard error. *p < 0.05; **p < 0.01; ***p < 0.001 compared with control intact simulation (Dunnett’s test)

To identify whether the experience decay parameter alone could account for differences in reversal learning performance we ran computational simulations. We first simulated the parameters for each group (Fig. 5), and then, using the control background, systematically altered all combinations of values from persistent psychosis subjects with impaired goal-directed action (Fig. 5, green bars). We analyzed the SRL1 stages looking at trials to criterion (Fig. 5A) as well as Win-stay use (Fig. 5B). A combination of increased experience decay and decreased inverse temperature was required to replicate the observed increases in trials to criterion and decreased Win-stay use. These results indicate that decreased inverse temperature in addition to increased experience decay is necessary to elicit performance deficits under the current task parameters.

### Psychiatric characteristics associated with behavioral performance

To identify which positive and negative symptoms were associated with differing aspects of behavioral performance, we used a multivariate general linear model (GLM), including the key behavioral outcomes, individual positive, and negative symptom scales, as well as age and chlorpromazine equivalent dose as control variables (Table 1). Decreased grandiosity (P5) and increased problems with abstract thinking (N5) were associated with decreased response bias for devaluation (i.e., impairment). This fits with group differences when split for intact and impaired performance (Table S7). For SRL measures, increases in poor rapport (N3) were associated with increased trials to criterion, decreased Win-stay and increased *rho* (experience decay) parameter scores. Increased hostility (P7) and stereotyped thinking (N7) were associated with decreased and increased Win-stay use, respectively. Increased stereotyped thinking (N7) also was associated with increases in the *beta* (inverse temperature) parameter. This indicates that increased problems with abstract thinking and poor rapport are key symptoms associated with the phenotypes observed in those with broad decision-making problems.

**Table 1:** Relationships between severity of symptoms and behavioral outcomes

		Devaluation		Serial-reversal learning					
		Valued action		Trials	Win-stay	Lose-shift	EWA model		
		Resp.	Bias				phi	rho	beta
P1	Delusions								
P2	Concept disorganization								
P3	Hallucinations								
P4	Excitement								
P5	Grandiosity		**+** (0.20) **						
P6	Suspiciousness/ persecution					**—** (0.18) *			
P7	Hostility				**—** (0.16) *				
N1	Blunt affect								
N2	Emotional withdrawal								
N3	Poor rapport			**+** (0.18) *	**—** (0.34) **			**+** (0.30) **	
N4	Passive/apathetic social withdrawal								
N5	Difficulty in abstract thinking		**—** (0.16) *						
N6	Lack of spontaneity/ flow of conversation								
N7	Stereotyped thinking				**+** (0.15) *				**+** (0.20) *

*Resp.*, response; *EWA*, experience-weighted attraction model; *phi*, learning rate; *rho*, experience decay; *beta*, inverse temperature. Data were analyzed using a multivariate GLM, including age and chlorpromazine equivalent dose (for which there were no significant relationships with behavior) and are expressed as direction of association (Partial Eta Squared). *p < 0.05; **p < 0.01

### Decreases in IQ do not underlie altered behavioral performance

Given that current IQ was lower in the persistent psychosis subjects with impaired goal-directed action compared with those with intact goal-directed action, we also examined persistent psychosis groups, with intact and impaired goal-directed action, matched for current and premorbid IQ (Table S11). This comparison indicated that alterations in trials to criterion, Win-stay, and experience decay were still present even when IQ was matched across groups.

## Discussion

Broad-decision-making impairments may reflect specific neurobiological pathology in a large proportion of those with psychosis, potentially leading to increased functional decline in these individuals. The present study expands on existing studies (Ceaser et al., 2008; Deserno et al., 2020; Pantelis et al., 1999; Reddy et al., 2016; Waltz & Gold, 2007; Weiler et al., 2009) to show that impairments in reversal learning may be driven by a specific group of individuals with psychosis, and these individuals also feature impaired goal-directed action. Furthermore, we observed some behavioral measures that were clearly specific to those with compromised goal-directed action (sluggish updating of experience weighting). Therefore, focusing specifically on the biological underpinnings of experience decay provides an avenue with which we can better understand the broad decision-making processes in this subgroup of individuals with psychosis.

### Decision-making deficits in people with persistent psychosis

Prior studies in those with persistent psychosis have observed deficits in goal-directed action and outcome devaluation (Morris et al., 2015; Morris et al., 2018; Pantelis et al., 2004). Because no differences in the ability to understand changes in value were observed, the performance changes were attributed to a deficit in the ability to encode causal actions (Morris et al., 2018). In contrast to these studies (Morris et al., 2015; Morris et al., 2018), we observed intact group level outcome devaluation, but the bimodal population of responding produced a weaker preference compared with control subjects. Similarly, reversal learning deficits have been observed consistently in persistent psychosis groups, often accompanied by decreased Win-stay strategy use (Ceaser et al., 2008; Deserno et al., 2020; Pantelis et al., 1999; Reddy et al., 2016; Waltz et al., 2013; Waltz & Gold, 2007; Weiler et al., 2009). Therefore, our results in those with psychosis support this prior work, but by assessing both decision-making processes we also were able to explore the relationship between goal-directed action impairments and serial-reversal learning phenotypes.

### Subgroup of those with psychosis feature broad impairments in decision-making processes

The bimodal profile we observed for response bias in those with psychosis provided an avenue to separate persistent psychosis subjects into two subgroups based on intact or impaired goal-directed action. This approach revealed that key measures of reversal learning performance were altered in those with impaired goal-directed action. In contrast, those with persistent psychosis and intact goal-directed action performed similarly to controls in their reversal learning. It has previously been reported that a proportion of those with persistent psychosis display impairments in reversal learning (Reddy et al., 2016), consistent with our findings. However, the former study separated subjects based on their discrimination learning capacity, whereas we observed no differences in the trials required to complete the initial discrimination in persistent psychosis subjects with impaired goal-directed action.

We hypothesized that if the associative striatum was dysfunctional, a neurobiological mechanism thought to underlie psychosis, then we would observe impaired performance in both tasks (Conn et al., 2020; Kesby et al., 2018). Our study demonstrates that impaired goal-directed action in those with persistent psychosis is accompanied by a specific reversal learning phenotype. Using computational modeling, we demonstrate that persistent psychosis subjects with impaired goal-directed action adapt to changing contingencies (i.e., reversals) more slowly than groups with intact goal-directed action. The EWA model was coded to reflect reversal learning-specific processes (den Ouden et al., 2013), with the experience decay parameter only impacting performance when contingencies were reversed. Differences in this parameter also may relate to striatal levels of dopamine (den Ouden et al., 2013), with preclinical studies suggesting that the associative striatum (dorsomedial striatum in rodents) is critical for evidence accumulation during learning (Yartsev et al., 2018). The increased experience decay values observed in persistent psychosis subjects with impaired goal-directed action suggests that they are less willing or able to update their prior understanding of the associated outcome values. Similar reversal learning deficits have been observed after treatment with methylphenidate (a dopamine transporter antagonist) in healthy individuals (Clatworthy et al., 2009), with those experiencing the greatest increase in dopamine in the associative striatum showing the greatest decline in reversal learning performance. Taken together, we suggest that these broad impairments in decision-making reflect altered information processing in the associative striatum of those with psychosis.

### Neurobiological mechanisms underlying impaired decision-making processes

Multiple neurobiological mechanisms could lead to broad decision-making impairments. Corticostriatal networks, including the striatal inputs from the anterior cingulate, orbitofrontal, and ventromedial cortices (Kesby et al., 2021), may be more severely compromised in a proportion of those with persistent psychosis leading to impairments in goal-directed action and experience updating. Studies in rodents highlight a complex role for the associative striatum in decision-making, with its primary action being the maintenance and selection of optimal decision-making strategies (Ragozzino, 2007). Most people with psychosis have increased associative striatal dopamine function (McCutcheon et al., 2018), and we observed that more than half of our participants with psychosis exhibited impaired goal-directed action. This aligns with observations of functional alterations in the caudate nucleus of those with psychosis during goal-directed action (Morris et al., 2015). However, there was no clear relationship between treatment-refractory (i.e., those treated with clozapine) cases and subgroup membership, even though treatment-refractory schizophrenia may occur without increases in striatal dopamine function (Demjaha et al., 2014). It is therefore possible that only those with the most severe levels of corticostriatal dysfunction, be that via increased dopamine or other neurobiological pathology (i.e., glutamatergic inputs from cortical areas), feature this specific pattern of decision-making deficits.

### Psychiatric characteristics in people with persistent psychosis and intact/impaired goal-directed action

The persistent psychosis subjects in our study had similar psychiatric characteristics overall regardless of capacity for goal-directed action; however, those with impaired goal-directed action exhibited a higher level of “difficulty in abstract thinking” and less severe ratings of grandiosity (Table S12). Increased difficulty in abstract thinking makes sense given the impairments in decision-making observed in this group. We speculate that decreased grandiosity may reflect some impairment in self-awareness and choice confidence arising from difficulties in understanding cause and effect. The most prominent association with reversal learning performance was “poor rapport,” a common negative symptom in psychosis subjects (Bobes et al., 2010). Although not focused on decision-making processes, decreased activation of the ventral striatum was observed in those with psychosis who had greater levels of “poor rapport” (Kumari et al., 2010). The ventral striatum is important in navigating reward feedback during reversal leaning (Kesby et al., 2021) and may explain this relationship. Clearly more work is required to understand the clinical and functional difficulties related to changes in the underlying neurobiology in these individuals.

## Conclusions

The current study has identified that a large proportion of people with persistent psychosis feature specific impairments in their decision-making capacity. Persistent psychosis subjects with impaired goal-directed action exhibited a decreased capacity to rapidly update their prior beliefs and associations in the face of changing contingencies. It is likely that these impairments would have significant functional implications in terms of planning and abstract thinking. These behavioral processes are sensitive to changes in associative striatal function suggesting common neurobiology may underlie the observed cognitive deficits.

## Supplementary information

ESM 1 (PDF 566 kb)
