# Supplementary material for: Impairments in goal-directed action and reversal learning in a proportion of individuals with psychosis
Source: Cogn Affect Behav Neurosci. 2022 Aug 2;22(6):1390–403. doi: 10.3758/s13415-022-01026-8 (PMC9622547; doi:10.3758/s13415-022-01026-8)
Supplement: Supplementary file 1 — (PDF 566 kb) [file 13415_2022_1026_MOESM1_ESM.pdf]

-- Supplementary Material --

Impairments in goal-directed action and reversal learning in a proportion of individuals with psychosis

Shuichi Suetani[1,2,3,4], Andrea Baker[1], Kelly Garner[2,5], Peter Cosgrove[1], Matilda Mackay-Sim[6], Dan Siskind[1,7,8], Graham K Murray[9,10,11], James G Scott[1,6,12] and James P Kesby[2,12*]

[1] Queensland Centre for Mental Health Research, Brisbane, QLD 4076, Australia.

[2] Queensland Brain Institute, The University of Queensland, Brisbane, QLD 4072, Australia.

[3] School of Medicine and Dentistry, Griffith University, Brisbane, QLD 4111, Australia.

[4] Institute for Urban Indigenous Health, Brisbane, QLD 4030, Australia.

[5] Centre for Human Brain Health, School of Psychology, University of Birmingham, Birmingham, United Kingdom.

[6] Metro North Mental Health, Royal Brisbane and Women's Hospital, Brisbane, QLD 4029, Australia.

[7] Metro South Addiction and Mental Health Services, Brisbane, QLD 4102, Australia.

[8] Faculty of Medicine, The University of Queensland, Brisbane, QLD 4072, Australia.

[9] Department of Psychiatry, University of Cambridge, Cambridge, UK.

[10] Cambridgeshire and Peterborough NHS Foundation Trust, Cambridge, UK.

[11] Institute for Molecular Bioscience, University of Queensland, Brisbane, Australia.

[12] QIMR Berghofer Medical Research Institute, Brisbane, QLD 4029, Australia.

* Corresponding author:

Dr. James Kesby

Queensland Brain Institute, The University of Queensland, Brisbane, QLD 4072, Australia.

Phone: +61 7 3346 6363 Fax: +61 7 3346 6301

Email: j.kesby@uq.edu.au ORCID: 0000-0002-5814-8062

SUPPLEMENTARY METHODS

Procedures and experimental design

We recruited people with psychosis from Metro North Hospital and Health Services, and Metro South Addiction and Mental Health Services in Brisbane, Australia. Healthy controls were recruited by using brochures to advertise the study. Participants were informed that they would receive $30 AUD for taking part in testing but could win a further $10 AUD based on the credits earned in the behavioral tasks. To avoid reduced compensation for those with psychosis and cognitive impairments, all participants were informed that they had passed the 'threshold' required for the full $10 compensation. Testing order was as follows: Substance Misuse Scale, outcome devaluation instrumental training, test of premorbid functioning (TOPF), devaluation testing, Wechsler Abbreviated Scale of Intelligence, second edition (WASI-II), reversal learning training stage, and reversal learning test stage.

Inclusion criteria

Those with persistent psychosis must have been diagnosed with a Persistent Psychotic Disorder (schizophrenia, schizoaffective disorder, bipolar I disorder, delusional disorder). Participants had no organic cause pf psychosis (i.e., epilepsy, intra-cranial pathology or HIV infection), and were between the ages of 18 and 50.

Serial reversal learning (SRL) task

For all stages of the reversal learning task, there were no limits on the time taken to respond. After selecting a stimulus, the outcome was presented on the screen for one second before the next trial was initiated. A running total of the 'credits' received was displayed in the bottom corner of the screen (participants were not aware of how much each credit was worth in monetary compensation). All stimulus pairs were binary images matched as closely as possible for white-black pixel ratio (see *Fig S2*), with all combinations being counterbalanced.

Initial training

Participants were shown the following instructions on the screen: *"Two pictures will appear on the screen. On each turn, use the joystick to choose one of these pictures. The computer will tell you what credits you earned for your choice. One of the pictures will get you a reward and the other will not. The pictures will change sides randomly, so be careful to select the correct one"*. They then began a deterministic discrimination with a single reversal, whereby every correct response was rewarded (outcome of 1) and incorrect responses were not (outcome of 0). The initial discrimination contingencies were reversed after 8 consecutive correct responses were made and both stages had to be completed within 200 trials (participants got up to two attempts using unique sets of stimuli).

Probabilistic serial reversal learning

After successfully completing the training stage, participants were administered a probabilistic SRL task. Two instruction screens were presented, with the first restating the instruction from the training test. The second informed the participants of the probabilistic contingencies: *"Unlike before, the correct picture will not always give you a reward and sometimes the wrong picture will give you a reward. Find out which picture earns the most credits. Stick with it even if it is sometimes wrong. At some point it may change so that the other picture earns more. Only start choosing the other picture when you are sure that the rule has changed"*.

The task consisted of 11 stages, each featuring the same pair of stimuli but varying in reward rate (probabilistic) and reward value (credits awarded). These included: initial discrimination (1 stage), initial reversal (1 stage), and serial reversal learning phase 1 (SRL1; 5 stages) and serial reversal learning phase 2 (SRL2; 4 stages). For the discrimination, initial reversal and SRL1 stages, the probabilistic reward contingencies were set at 80/20, meaning that the target stimulus was rewarded 80% of the time, whereas the non-target stimulus was rewarded only 20% of the time. The reward outcomes included 1 credit for a rewarded trial and 0 credits for a non-rewarded trial.

For the SRL2 stages, the probabilistic reward contingencies were set at 80/40 to increase the task difficulty, meaning that the target stimulus was rewarded 80% of the time, whereas the non-target stimulus was rewarded 40% of the time. The reward outcomes were 2 or 6 credits for a rewarded trial (of equal probability) and 0 credits for a non-rewarded trial. The addition of variable reward values was included to analyze whether the strategy that a participant used was biased when receiving greater rewards on the preceding trial. Criterion for progressing to the next stage was 6 correct responses in a row. The test ended once the participant completed all 11 stages, or once 500 trials were completed. SRL1 and SRL2 trials to criterion were only included in analyses if at least 2 stages had been completed. All trials (completion of stage or not) were included in all other analyses.

Reversal learning performance measures and strategies

There are multiple measures of performance that can be quantified in reversal learning tasks. These include, but are not limited to, total trials to criterion, perseveration (number of errors in the first 6 trials after a reversal), and response rates (total, or for correct and incorrect responses). Other measures allow for detailed inspections of choice strategy, including whether a subject selects the same stimulus after attaining a reward (Win-stay) or whether they select the other stimulus (Win-shift). Similar strategies were calculated after losses, including whether the subject selected the same stimulus after a non-rewarded trial (Lose-stay) or selected the other stimulus (Lose-shift). These were calculated as the proportion of each strategy relative to the trials in which that strategy could be used (i.e., P.Win-stay = Total number of times the same stimulus was selected after a rewarded trial/total rewarded trials). All values were calculated for each individual stage, as well as across the SRL1 and SRL2 stages (inclusive of all combined trials).

Serial reversal learning exclusions

One participant (a male in the persistent psychosis group) failed to successfully complete the training stages after two attempts. Data from this participant were included in the outcome devaluation data but not for reversal learning or intact/impaired analyses.

SUPPLEMENTARY TABLES

Table S1. Psychiatric characteristics and symptom assessments in those with psychosis.

**Group**		**Persistent psychosis (N = 45)**
Diagnosis (% Naff, Aff, Other)		51%, 42%, 7%
**Medications**		
Dose (chlorpromazine equivalent)		481.0 (411.6)
Number		4.07 (2.85)
Anxiety (% Yes)		15.6%
Mood stabilisers (% Yes)		22.2%
Antidepressants (% Yes)		36.6%
Substance dependence (% Yes)		11.1%
**PANSS**		
P1	Delusions	2.22 (1.61)
P2	Conceptual disorganisation	1.58 (1.22)
P3	Hallucinatory behavior	2.51 (1.60)
P4	Excitement	1.40 (0.86)
P5	Grandiosity	2.11 (1.35)
P6	Suspiciousness/persecution	2.47 (1.49)
P7	Hostility	1.22 (0.60)
**Positive Scale Total**		**13.51 (5.69)**
N1	Blunted affect	2.87 (1.55)
N2	Emotional withdrawal	1.93 (1.25)
N3	Poor rapport	1.69 (1.10)
N4	Passive social withdrawal	1.84 (1.15)
N5	Difficulty in abstract thinking	3.51 (1.16)
N6	Lack of spontaneity/flow	1.80 (1.25)
N7	Stereotyped thinking	1.89 (1.17)
**Negative Scale Total**		**15.53 (6.07)**
G1	Somatic concern	2.38 (1.13)
G2	Anxiety	2.64 (1.42)
G3	Guilt feelings	1.69 (1.20)
G4	Tension	2.02 (0.94)
G5	Mannerisms and posturing	1.58 (0.99)
G6	Depression	2.42 (1.45)
G7	Motor retardation	1.56 (1.12)
G8	Uncooperativeness	1.18 (0.58)
G9	Unusual thought content	1.69 (1.00)
G10	Disorientation	2.33 (0.80)
G11	Poor attention	1.60 (1.10)
G12	Lack of judgment and insight	3.09 (1.59)
G13	Disturbance of volition	1.36 (0.74)
G14	Poor impulse control	1.18 (0.58)
G15	Preoccupation	1.84 (1.33)
G16	Active social avoidance	2.04 (1.30)
**General Psychopathology Scale Total**		**30.60 (7.39)**
**PANSS Total**		**59.64 (15.73)**

Medication classifications: anxiety (Pregabalin, benzodiazepines), mood stabilisers (lithium), antidepressants (selective serotonin reuptake inhibitors [SSRIs], monoamine oxidase inhibitors, Mirtazapine), and substance dependence (Naltrexone, buprenorphine, Varenicline). *NAff*, nonaffective disorder; *Aff*, affective disorder; *AP*, antipsychotic. The data are expressed as mean (± standard deviation) where applicable.

Table S2. Demographics, IQ and substance use characteristics in persistent psychosis.

Group		Controls (N = 34)	Persistent psychosis (N = 45)	*F*/χ^2	*p*	
**Demographic characteristics**						
Age (years)		32.4 (9.9)	31.0 (8.8)	0.39	0.536	
Education (years)		14.9 (2.7)	11.5 (2.0)	40.53	<0.001	***
Gender (% male)		44.1%	73.3%	6.93	0.008	**
Ethnicity (% Caucasian)		85.29%	66.7%	3.55	0.059	
**IQ test scores**						
TOPF (ss)		112.2 (8.9)	98.0 (14.4)	25.43	<0.001	***
WASI-II (FISQ-2)		110.1 (10.0)	89.5 (16.9)	40.33	<0.001	***
**Substance use characteristics**						
Alcohol	Lifetime	97.1%	91.1%	1.16	0.282	
	28d freq	2.26 (1.33)	1.00 (1.26)	18.55	<0.001	***
Cannabinoids	Lifetime	61.8%	82.2%	4.15	0.042	*
	28d freq	0.32 (1.01)	0.53 (1.27)	0.63	0.431	
Nicotine	Lifetime	50.0%	84.4%	10.86	<0.001	***
	28d freq	0.85 (2.02)	3.87 (2.84)	27.67	<0.001	***
Caffeine	Lifetime	82.4%	95.6%	3.71	0.054	
	28d freq	4.00 (2.23)	4.18 (2.20)	0.13	0.724	
Amphetamines	Lifetime	8.8%	53.3%	17.06	<0.001	***
	28d freq	0.00 (0.00)	0.09 (0.47)	1.22	0.272	
Ecstasy	Lifetime	23.5%	55.6%	8.17	0.004	**
	28d freq	0.03 (0.17)	0.04 (0.30)	0.69	0.793	
Opiates	Lifetime	2.9%	17.8%	4.22	0.040	*
	28d freq	0.00 (0.00)	0.00 (0.00)	-	-	
Benzodiazepines	Lifetime	5.9%	24.4%	4.85	0.028	*
	28d freq	0.00 (0.00)	0.00 (0.00)	-	-	
Other	Lifetime	26.5%	35.6%	0.74	0.390	
	28d freq	0.06 (0.34)	0.00 (0.00)	0.88	0.418	
Volatile	Lifetime	2.9%	17.8%	4.22	0.040	*
	28d freq	0.00 (0.00)	0.00 (0.00)	-	-	

28 day frequency (28d freq) was scored using the following criteria; 0 = no use, 1 = once in 28 days, 2 = 2-3x in 28 days, 3 = 1-2x/week, 4 = 3-6x/week, 5 = daily, or 6 = multiple uses daily. The data are expressed as mean (± standard deviation) where applicable. TOPF, test of premorbid functioning; ss, standard score; WASI-II, Wechsler Abbreviated Scale of Intelligence – 2nd edition; FSIQ-2, Full-Scale IQ. *$p<0.05$, **$p<0.01$, ***$p<0.001$.

Table S3. Outcome-specific devaluation in control subjects those with persistent psychosis.

Group	Controls (N = 34)	Persistent psychosis (N = 44)	F/χ^2	p	
**Instrumental training**					
Total trials to criterion	6.82 (2.38)	7.39 (2.75)	0.90	0.345	
Total correct trials	6.44 (1.62)	6.59 (1.65)	0.16	0.689	
Response rate (s)	1.45 (0.63)	2.05 (1.37)	5.56	0.021	*
Correct response rate (s)	1.34 (0.63)	1.73 (1.17)	3.09	0.083	
**Devaluation rating changes**					
Valued stimulus	1.03 (1.45) [aaa]	0.93 (1.39) [aaa]	0.09	0.763	
Devalued stimulus	-2.06 (2.03)	-1.07 (2.18)	4.20	0.044	*
Irrelevant stimulus	0.00 (1.30)	-0.07 (1.81)	0.03	0.853	
Motivation to earn credits	0.00 (0.98)	-0.14 (1.19)	0.29	0.591	
**Outcome-specific devaluation**					
Valued response ratio	0.85 (0.33) [aaa]	0.67 (0.29) [aaa]	6.86	0.011	*
Devalued response ratio	0.15 (0.33)	0.33 (0.29)	6.86	0.011	*
Valued response rate (/s)	2.86 (1.42) [aaa]	1.83 (1.12) [aaa]	12.75	<0.001	***
Devalued response rate (/s)	0.59 (1.37)	0.77 (0.73)	0.52	0.472	
**Probe questions**					
Average correct responses	1.44 (0.79)	1.27 (0.85)	0.81	0.371	

The data are expressed as mean (± standard deviation) where applicable. *p<0.05, ***p<0.001.

[aaa]p<0.001 valued outcome compared with equivalent devalued outcome (paired t-test within group).

Table S4. Serial reversal learning in control subjects those with persistent psychosis.

Group	Controls (N = 34) †	Persistent psychosis (N = 44) †	*F*/χ^2	*p*	
**Trials to criterion**					
Initial discrimination	19.85 (46.53)	18.18 (14.09)	0.05	0.822	
First reversal	12.71 (6.95)	25.45 (35.11)	4.34	0.041	*
SRL1	14.06 (9.65)	20.87 (18.59)	3.76	0.056	
SRL2	22.64 (19.11)	30.45 (27.35)	1.86	0.177	
**Strategy use**					
SRL1 Win-stay	0.97 (0.08)	0.88 (0.15)	9.00	0.004	**
SRL1 Lose-shift	0.55 (0.26)	0.59 (0.20)	0.80	0.374	
SRL2 Win-stay	0.90 (0.14)	0.82 (0.16)	5.39	0.023	*
SRL2 Win-stay 2	0.88 (0.16)	0.81 (0.18)	3.49	0.066	
SRL2 Win-stay 6	0.92 (0.16)	0.82 (0.17)	6.22	0.015	*
SRL2 Lose-shift	0.52 (0.25)	0.57 (0.25)	0.96	0.330	
**Computational modelling**					
EWA *phi*	0.22 (0.19)	0.12 (0.13)	7.19	0.009	**
EWA *rho*	0.22 (0.19)	0.34 (0.27)	4.57	0.036	*
EWA *beta*	2.53 (0.78)	2.02 (0.73)	8.56	0.005	**

The data are expressed as mean (± standard deviation) where applicable. † for SRL2 outcomes, N = 33 for control and N = 41 for persistent psychosis groups. *p<0.05, **p<0.01.

Table S5. Demographics, IQ and substance use characteristics for control subjects split for intact or impaired goal-directed action.

Group		Control	Control
**Goal-directed action**		**intact (N = 28)**	**impaired (N = 6)**
**Demographics**			
Age (years)		32.3 (10.0)	32.5 (10.4)
Education (years)		14.7 (2.8)	15.5 (2.0)
Gender (% male)		35.7%	83.3%
Ethnicity (% Caucasian)		89.3%	66.7%
**IQ test scores**			
TOPF (ss)		111.5 (9.4)	115.2 (6.4)
WASI-II (FISQ-2)		110.1 (10.7)	110.3 (5.9)
**Substance use**			
Alcohol	Lifetime	100.0%	83.3%
	28d freq	2.25 (1.29)	2.33 (1.63)
Cannabinoids	Lifetime	64.3%	50.0%
	28d freq	0.21 (0.63)	0.83 (2.04)
Nicotine	Lifetime	50.0%	50.0%
	28d freq	0.82 (1.96)	1.00 (2.45)
Caffeine	Lifetime	82.1%	83.3%
	28d freq	3.93 (2.26)	4.33 (2.25)
Amphetamines	Lifetime	7.1%	16.7%
	28d freq	—	—
Ecstasy	Lifetime	21.4%	33.3%
	28d freq	0.04 (0.19)	—
Opiates	Lifetime	3.6%	0.0%
	28d freq	—	—
Benzodiazepines	Lifetime	7.1%	0.0%
	28d freq	—	—
Other	Lifetime	25.0%	33.3%
	28d freq	0.07 (0.38)	—
Volatile	Lifetime	0.0%	16.7%
	28d freq	—	—

28 day frequency (28d freq) was scored using the following criteria; 0 = no use, 1 = once in 28 days, 2 = 2-3x in 28 days, 3 = 1-2x/week, 4 = 3-6x/week, 5 = daily, or 6 = multiple uses daily. The data are expressed as mean (± standard deviation) where applicable. TOPF, test of premorbid functioning; ss, standard score; WASI-II, Wechsler Abbreviated Scale of Intelligence – 2nd edition; FSIQ-2, Full-Scale IQ.

Table S6. Demographics, IQ and substance use characteristics for those with persistent psychosis split for intact or impaired goal-directed action.

Group		Control	Persistent psychosis	Persistent psychosis	F/χ^2	*p*
**Goal-directed action**		**intact (N = 28)**	**intact (N = 18)**	**impaired (N = 25)**		
**Demographic characteristics**						
Age (years)		32.3 (10.0)	31.6 (9.6)	31.2 (8.4)	0.10	0.902
Education (years)		14.7 (2.8)	12.4 (1.5) ##	10.9 (2.1) ###	18.75	<0.001
Gender (% male)		35.7%	77.8%	68.0%	9.61	0.008
Ethnicity (% Caucasian)		89.3%	83.3%	56.0%	8.74	0.013
**IQ test scores**						
TOPF (ss)		111.5 (9.4)	103.8 (9.8)	94.5 (15.1) ###	13.81	<0.001
WASI-II (FISQ-2)		110.1 (10.7)	98.8 (14.9) #	83.5 (14.5) **	26.62	<0.001
**Substance use characteristics**						
Alcohol	Lifetime	100.0%	88.9%	96.0%	3.35	0.188
	28d freq	2.25 (1.29)	1.33 (1.46)	0.84 (1.11) ###	8.33	0.001
Cannabinoids	Lifetime	64.3%	72.2%	88.0%	4.00	0.135
	28d freq	0.21 (0.63)	0.67 (1.57)	0.48 (1.08)	1.00	0.375
Nicotine	Lifetime	50.0%	83.3%	84.0% #	9.26	0.010
	28d freq	0.82 (1.96)	3.33 (2.91) ##	4.08 (2.86) ###	11.72	<0.001
Caffeine	Lifetime	82.1%	94.4%	96.0%	3.36	0.187
	28d freq	3.93 (2.26)	4.17 (2.28)	4.04 (2.21)	0.06	0.940
Amphetamines	Lifetime	7.1%	44.4% #	60.0% #	17.08	<0.001
	28d freq	0.00 (0.00)	0.00 (0.00)	0.16 (0.62)	1.51	0.229
Ecstasy	Lifetime	21.4%	61.1% #	52.0%	8.57	0.014
	28d freq	0.04 (0.19)	0.11 (0.47)	0.00 (0.00)	0.94	0.395
Opiates	Lifetime	3.6%	16.7%	20.0%	3.57	0.168
	28d freq	0.00 (0.00)	0.00 (0.00)	0.00 (0.00)	-	-
Benzodiazepines	Lifetime	7.1%	38.9% #	16.0%	7.52	0.023
	28d freq	0.00 (0.00)	0.00 (0.00)	0.00 (0.00)	-	-
Other	Lifetime	25.0%	44.4%	28.0%	2.10	0.350
	28d freq	0.07 (0.38)	0.00 (0.00)	0.00 (0.00)	0.76	0.470
Volatile	Lifetime	0.0%	22.2% #	16.0%	6.276	0.043
	28d freq	0.00 (0.00)	0.00 (0.00)	0.00 (0.00)	-	-

28 day frequency (28d freq) was scored using the following criteria; 0 = no use, 1 = once in 28 days, 2 = 2-3x in 28 days, 3 = 1-2x/week, 4 = 3-6x/week, 5 = daily, or 6 = multiple uses daily. The data are expressed as mean (± standard deviation) where applicable. TOPF, test of premorbid functioning; ss, standard score; WASI-II, Wechsler Abbreviated Scale of Intelligence – 2nd edition; FSIQ-2, Full-Scale IQ. ***p*<0.01 compared with all groups; #*p*<0.05, ##*p*<0.01, ###*p*<0.001 compared with Controls.

Table S7. Psychiatric characteristics and symptom assessments for those with persistent psychosis split for intact or impaired goal-directed action.

Group		Persistent psychosis	Persistent psychosis	F/χ^2	p	
**Goal-directed action**		**intact (N = 18)**	**impaired (N = 25)**			
Diagnosis (% Naff, Aff, Other)		56%, 44%, 0%	44%, 39%, 17%	4.51	0.105	
**Medications**						
Dose (chlorpromazine equivalent)		424.8 (225.9)	521.8 (513.8)	0.56	0.458	
Number		3.61 (2.97)	4.48 (2.80)	0.96	0.334	
Anxiety (% Yes)		11.1%	20.0%	0.61	0.436	
Mood stabilisers (% Yes)		27.8%	16.0%	0.88	0.349	
Antidepressants (% Yes)		33.3%	40.0%	0.20	0.655	
Substance dependence (% Yes)		16.7%	8.0%	0.77	0.382	
**PANSS**						
P1	Delusions	2.39 (1.72)	2.20 (1.58)	0.14	0.711	
P2	Conceptual disorganisation	1.72 (1.41)	1.52 (1.12)	0.28	0.603	
P3	Hallucinatory behaviour	2.44 (1.85)	2.60 (1.47)	0.09	0.761	
P4	Excitement	1.61 (1.04)	1.28 (0.74)	1.50	0.227	
P5	Grandiosity	2.72 (1.53)	1.64 (1.08)	7.46	0.009	**
P6	Suspiciousness/persecution	2.39 (1.58)	2.56 (1.50)	0.13	0.720	
P7	Hostility	1.06 (0.24)	1.36 (0.76)	2.71	0.108	
**Positive Scale Total**		**14.33 (6.13)**	**13.16 (5.59)**	**0.43**	**0.518**	
N1	Blunted affect	2.56 (1.62)	3.08 (1.44)	1.25	0.270	
N2	Emotional withdrawal	1.67 (1.14)	2.08 (1.29)	1.19	0.283	
N3	Poor rapport	1.39 (1.04)	1.84 (1.07)	1.91	0.174	
N4	Passive social withdrawal	1.89 (1.28)	1.88 (1.09)	0.00	0.981	
N5	Difficulty in abstract thinking	2.94 (1.00)	3.88 (1.01)	9.03	0.005	**
N6	Lack of spontaneity/flow	1.39 (1.14)	2.00 (1.15)	2.95	0.093	
N7	Stereotyped thinking	1.94 (1.30)	1.72 (1.02)	0.40	0.530	
**Negative Scale Total**		**13.78 (6.30)**	**16.48 (5.61)**	**2.19**	**0.146**	
G1	Somatic concern	2.61 (1.20)	2.24 (1.09)	1.12	0.296	
G2	Anxiety	2.56 (1.42)	2.84 (1.40)	0.42	0.518	
G3	Guilt feelings	1.83 (1.38)	1.56 (1.08)	0.53	0.471	
G4	Tension	1.89 (0.96)	2.20 (0.91)	1.16	0.288	
G5	Mannerisms and posturing	1.50 (0.86)	1.64 (1.11)	0.20	0.658	
G6	Depression	2.56 (1.54)	2.44 (1.42)	0.07	0.801	
G7	Motor retardation	1.33 (1.03)	1.60 (1.00)	0.73	0.399	
G8	Uncooperativeness	1.00 (0.00)	1.24 (0.66)	2.34	0.134	
G9	Unusual thought content	1.89 (1.13)	1.60 (0.91)	0.86	0.360	
G10	Disorientation	2.17 (0.86)	2.52 (0.71)	2.17	0.149	
G11	Poor attention	1.39 (1.24)	1.80 (1.00)	1.44	0.237	
G12	Lack of judgment and insight	2.78 (1.59)	3.24 (1.64)	0.85	0.362	
G13	Disturbance of volition	1.50 (0.92)	1.28 (0.61)	0.88	0.353	
G14	Poor impulse control	1.22 (0.65)	1.16 (0.55)	0.12	0.736	
G15	Preoccupation	1.72 (1.36)	1.84 (1.21)	0.09	0.767	
G16	Active social avoidance	1.83 (1.42)	2.20 (1.22)	0.82	0.371	
**General Psychopathology Scale Total**		**29.78 (9.72)**	**31.40 (5.53)**	**0.48**	**0.491**	
**PANSS Total**		**57.89 (19.70)**	**61.04 (12.81)**	**0.40**	**0.528**	

Medication classifications: anxiety (Pregabalin, benzodiazepines), mood stabilisers (lithium), antidepressants (selective serotonin reuptake inhibitors [SSRIs], monoamine oxidase inhibitors, Mirtazapine), and substance dependence (Naltrexone, buprenorphine, Varenicline). NAff, nonaffective disorder; Aff, affective disorder. The data are expressed as mean (± standard deviation) where applicable. ***p*<0.01.

Table S8. Outcome-specific devaluation in controls and those with persistent psychosis split for intact or impaired goal-directed action.

**Group**	**Control**	**Persistent psychosis**	**Persistent psychosis**	***F/χ²***	***p***
**Goal-directed action**	**intact (N = 28)**	**intact (N = 18)**	**impaired (N = 25)**		
**Instrumental training**					
Total trials to criterion	6.21 (0.63)	7.00 (2.87)	7.44 (2.50)	2.31	0.107
Total correct trials	6.07 (0.38)	6.33 (1.41)	6.68 (1.77)	1.47	0.237
Response rate (s)	1.48 (0.64)	2.03 (1.15)	1.80 (0.86)	2.38	0.100
Correct response rate (s)	1.38 (0.67)	1.89 (1.20)	1.44 (0.65)	2.27	0.111
**Devaluation rating changes**					
Valued stimulus	1.14 (1.51) [aaa]	0.72 (1.02) [aaa]	1.04 (1.62) [a]	0.48	0.622
Devalued stimulus	-2.21 (1.93)	-2.22 (1.99)	-0.28 (2.01) **	7.80	0.001
Irrelevant stimulus	0.04 (1.43)	-0.28 (2.24)	0.08 (1.50)	0.27	0.766
Motivation to earn credits	0.14 (0.89)	0.28 (0.75)	-0.48 (1.36)	3.43	0.038
**Outcome-specific devaluation**					
Valued response ratio	0.99 (0.03) [aaa]	0.98 (0.05) [aaa]	0.45 (0.17) ***	47.05	<0.001
Devalued response ratio	0.01 (0.03)	0.02 (0.05)	0.55 (0.17) ***	112.91	<0.001
Valued response rate (/s)	3.26 (1.03) [aaa]	2.88 (0.77) [aaa]	1.08 (0.66) ***	203.38	<0.001
Devalued response rate (/s)	0.02 (0.06)	0.07 (0.16)	1.27 (0.54) ***	203.38	<0.001
**Probe questions**					
Average correct responses	1.61 (0.69)	1.56 (0.51)	1.12 (0.97)	3.05	0.054

The data are expressed as mean (± standard deviation) where applicable. ***p*<0.01, ****p*<0.001 compared with all groups; [a]*p*<0.05, [aaa]*p*<0.001 valued outcome compared with equivalent devalued outcome (paired t-test within group).

Table S9. Outcome-specific devaluation in a subgroup of control subjects **matched for rating changes towards the devalued stimuli** with persistent psychosis subjects with impaired goal-directed action.

**Group**	**Control**	**Persistent psychosis**	***F/χ²***	***p***	
**Goal-directed action**	**intact (N = 15)**	**impaired (N = 25)**			
**Instrumental training**					
Total trials to criterion	6.20 (0.77)	7.44 (2.50)	3.46	0.071	
Total correct trials	6.13 (0.52)	6.68 (1.77)	1.35	0.253	
Response rate (s)	1.40 (0.67)	1.80 (0.86)	2.44	0.127	
Correct response rate (s)	1.39 (0.67)	1.44 (0.65)	0.04	0.840	
**Devaluation rating changes**					
Valued stimulus	1.20 (1.70)	1.04 (1.62)	0.09	0.768	
Devalued stimulus	***-0.93 (1.03)***	***-0.28 (2.01)***	***1.36***	***0.251***	
Irrelevant stimulus	0.27 (0.88)	0.08 (1.50)	0.19	0.664	
Motivation to earn credits	0.40 (1.12)	-0.48 (1.36)	4.46	0.041	*
**Outcome-specific devaluation**					
Valued response ratio	0.99 (0.04) [aaa]	0.45 (0.17) [aaa]	142.46	<0.001	***
Devalued response ratio	0.01 (0.04)	0.55 (0.17)	142.46	<0.001	***
Valued response rate (/s)	2.88 (0.73) [a]	1.08 (0.66)	63.47	<0.001	***
Devalued response rate (/s)	0.02 (0.07)	1.27 (0.54)	79.83	<0.001	***
**Probe questions**					
Average correct responses	1.67 (0.62)	1.12 (0.97)	3.81	0.058	

The data are expressed as mean (± standard deviation) where applicable.

*p<0.05, ***p<0.001. [a]p<0.05, [aaa]p<0.001 valued outcome compared with equivalent devalued outcome (paired t-test within group).

Table S10. Serial reversal learning in controls and those with persistent psychosis split for intact or impaired goal-directed action.

**Group**	**Control**	**Persistent psychosis**	**Persistent psychosis**	***F/χ²***	***p***
**Goal-directed action**	**intact (N = 28) †**	**intact (N = 18)**	**impaired (N = 25) †**		
**Trials to criterion**					
Initial discrimination	21.89 (51.16)	17.22 (13.74)	19.12 (14.79)	0.11	0.897
First reversal	12.29 (6.83)	17.78 (11.89)	31.60 (44.89) #	3.34	0.042
SRL1	14.59 (10.48)	13.57 (8.21)	26.51 (22.49) *****	5.12	0.009
SRL2	20.08 (13.33)	21.74 (9.96)	38.60 (35.54) #	4.46	0.016
**Strategy use**					
SRL1 Win-stay	0.96 (0.09)	0.95 (0.09)	0.83 (0.16) ******	9.57	<0.001
SRL1 Lose-shift	0.55 (0.26)	0.62 (0.20)	0.59 (0.18)	0.73	0.484
SRL2 Win-stay	0.91 (0.13)	0.84 (0.12)	0.78 (0.19) #	4.07	0.022
SRL2 Win-stay 2	0.89 (0.14)	0.81 (0.15)	0.80 (0.20)	2.47	0.092
SRL2 Win-stay 6	0.92 (0.17)	0.87 (0.13)	0.77 (0.19) #	5.02	0.009
SRL2 Lose-shift	0.52 (0.23)	0.57 (0.25)	0.60 (0.23)	0.73	0.485
**Computational modelling**					
EWA *phi*	0.22 (0.21)	0.10 (0.10) #	0.12 (0.13)	4.18	0.019
EWA *rho*	0.23 (0.20)	0.23 (0.17)	0.43 (0.29) ******	5.55	0.006
EWA *beta*	2.54 (0.81)	2.10 (0.59)	1.88 (0.70) ##	5.80	0.005

The data are expressed as mean (± standard deviation) where applicable. **†** for SRL2 outcomes, N = 27 for control intact and N = 22 for persistent psychosis impaired groups. **p*<0.05, ***p*<0.01 compared with all groups; #*p*<0.05, ##*p*<0.01 compared with Controls.

Table S11. Behavioral differences in those with persistent psychosis split for intact or impaired goal-directed action and **matched for IQ**.

**Group**	**Persistent psychosis**	**Persistent psychosis**	***F***	***p***	
**Goal-directed action**	**intact (N = 17)**	**impaired (N = 17)**			
**General characteristics**					
Age (years)	32.2 (9.5)	31.2 (9.9)	0.10	0.753	
Dose (CPZ equivalent)	449.8 (205.6)	575.6 (608.6)	0.65	0.425	
**IQ test scores**					
TOPF (ss)	103.3 (9.8)	97.3 (14.8)	1.95	0.172	
WASI-II (FISQ-2)	96.6 (11.8)	91.5 (9.6)	1.89	0.179	
**Outcome-specific devaluation**					
Valued response ratio	0.97 (0.05)	0.49 (0.15)	160.27	<0.001	***
Valued response rate (/s)	2.93 (0.76)	1.24 (0.7)	44.62	<0.001	***
**Serial reversal learning**					
SRL1 (trials to criterion)	13.88 (8.35)	26.13 (20.46)	5.22	0.029	*
SRL1 Win-stay	0.95 (0.09)	0.83 (0.16)	7.77	0.009	**
SRL1 Lose-shift	0.63 (0.2)	0.63 (0.16)	0.00	0.991	
**Computational modelling**					
EWA phi	0.16 (0.17)	0.09 (0.06)	0.55	0.463	
EWA rho	0.38 (0.21)	0.48 (0.28)	4.59	0.040	*
EWA beta	2.07 (0.56)	1.96 (0.71)	1.87	0.181	

The data are expressed as mean (± standard deviation) where applicable. CPZ, chlorpromazine; TOPF, test of premorbid functioning; ss, standard score; WASI-II, Wechsler Abbreviated Scale of Intelligence – 2nd edition; FSIQ-2, Full-Scale IQ. *p<0.05, **p<0.01, ***p<0.001.

SUPPLEMENTARY FIGURES

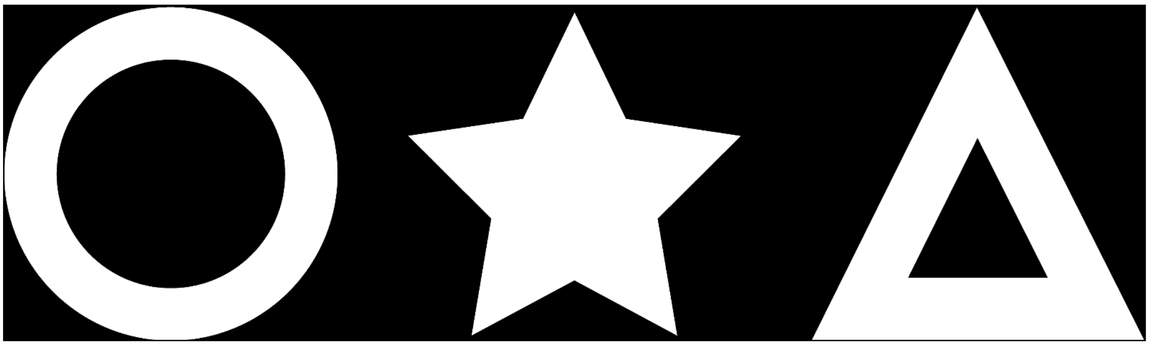

Figure S1. Stimuli used for outcome-specific devaluation tokens. All stimuli were matched for white:black pixel ratio.

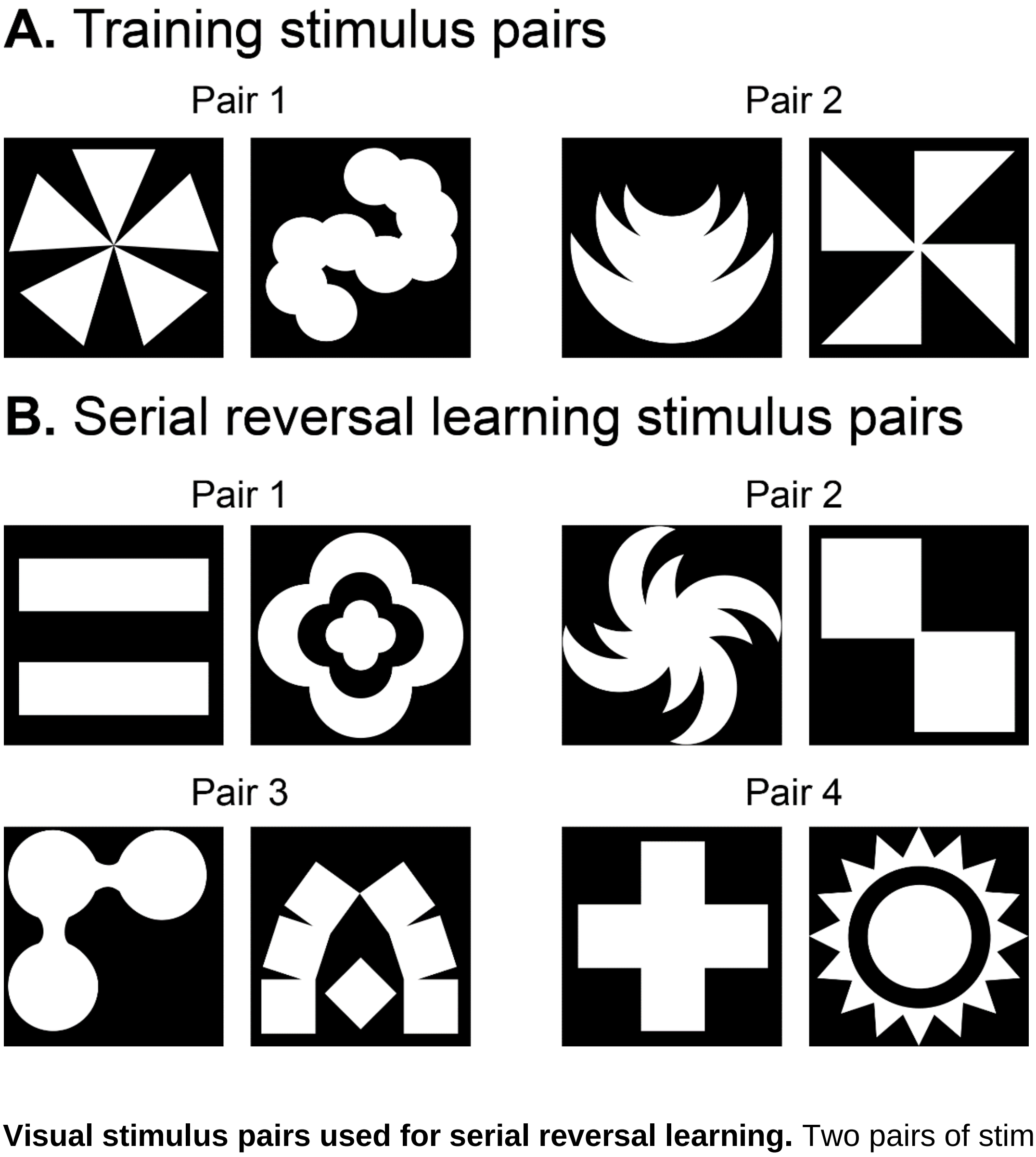

Figure S2. Visual stimulus pairs used for serial reversal learning. Two pairs of stimuli were used for the training stage (**A**). Four pairs of stimuli were used for the serial reversal learning test stage (**B**). Pair sets and stimuli assigned as the initial 'correct' stimuli were counterbalanced between groups. All stimuli were matched for white:black pixel ratio.

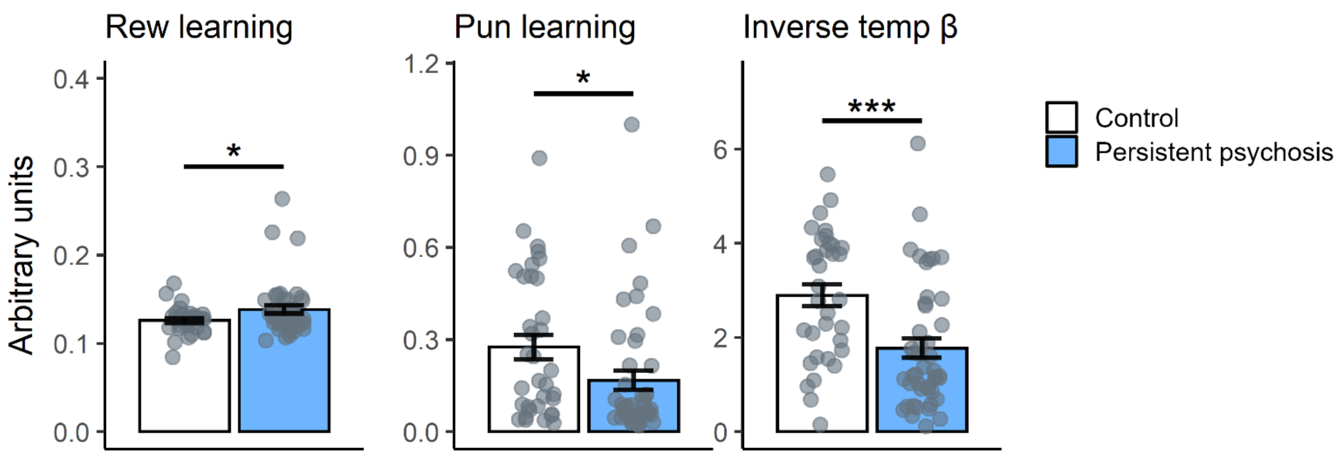

Figure S3. Reward/punishment model for reversal learning in those with persistent psychosis. Comparison of fitted reward/punishment model parameters for reversal learning performance in healthy controls and those with persistent psychosis. Reward (Rew) and punishment (Pun) learning are inverted (1 – learning rate) so that directionality is consistent with those of the EWA model. Differences in computational modeling parameters were observed for all parameters. Higher reward learning in those with persistent psychosis indicates a bias towards past wins, and decreased punishment learning indicates a bias towards recent losses. Decreased inverse temperature (temp) values in those with persistent psychosis reflect less deterministic or more exploratory decision-making. Data are displayed as the mean ± standard error. *p<0.05, ***p<0.001.
